# Readmission within three months after inpatient geriatric care—Incidence, diagnosis and associated factors in a Swedish cohort

**DOI:** 10.1371/journal.pone.0248972

**Published:** 2021-03-22

**Authors:** Carl Willers, Anne-Marie Boström, Lennart Carlsson, Anton Lager, Rikard Lindqvist, Elisabeth Rydwik

**Affiliations:** 1 Division of Physiotherapy, Department of Neurobiology, Care Sciences and Society, Karolinska Institutet, Stockholm, Sweden; 2 Region Stockholm, FOU nu, Research and Development Center for the Elderly, Stockholm, Sweden; 3 Division of Nursing, Department of Neurobiology, Care Sciences and Society, Karolinska Institutet, Stockholm, Sweden; 4 Karolinska University Hospital, Theme Aging, Stockholm, Sweden; 5 R&D Unit, Stockholms Sjukhem, Stockholm, Sweden; 6 Division of Family Medicine and Primary Care, Department of Neurobiology, Care Sciences and Society, Karolinska Institutet, Stockholm, Sweden; 7 Region Stockholm, Centre for Epidemiology and Community Medicine, Stockholm, Sweden; 8 Department of Global Public Health, Karolinska Institutet, Stockholm, Sweden; 9 Department of Learning, Informatics, Management, and Ethics, Karolinska Institutet, Stockholm, Sweden; 10 Medical Unit for Aging, Health and Function, Function Allied Health Professionals, Karolinska University Hospital, Stockholm, Sweden; University of Alberta, CANADA

## Abstract

**Introduction:**

Readmissions are very costly, in monetary terms but also for the individual patient’s safety and health. Only by understanding the reasons and drivers of readmissions, it is possible to ensure quality of care and improve the situation. The aim of this study was to assess inpatient readmissions during the first three months after discharge from geriatric inpatient care regarding main diagnosis and frequency of readmission. Furthermore, the aim was to analyze association between readmission and patient characteristics including demography and socioeconomics, morbidity, physical function, risk screening and care process respectively.

**Methods:**

The study includes all individuals admitted for inpatient care at three geriatric departments operated by the Stockholm region during 2016. Readmission after discharge was studied within three different time intervals; readmission within 10 days after discharge, within 11–30 days and within 31–90 days, respectively. Main diagnosis at readmission was assessed.

**Results:**

One fourth of the individuals discharged from inpatient geriatric care was readmitted during the first three months after discharge. The most common main diagnoses for readmission were heart failure, chronic obstructive pulmonary disease and pneumonia. Statistically significant risk factors for readmission included age, sex, number of diagnoses at discharge, and to some extent polypharmacy and destination of discharge.

**Conclusions:**

Several clinical risk factors relating to physical performance and vulnerability were associated with risk of readmission. Socioeconomic information did not add to the predictability. To enable reductions in readmission rates, proactive monitoring of frail individuals afflicted with chronic conditions is necessary, and an integrated perspective including all stakeholders involved is crucial.

## Introduction

Patients in need of geriatric care are often afflicted with several medical conditions, with associated different treatment regimens supposed to run in parallel [1]. These patients are often fragile with little or no own ability to actively navigate the often complex healthcare system [2]. Geriatric care in Stockholm is a responsibility both of the region (which finances or finances and operates all inpatient, specialized outpatient and primary care) as well as of the local municipalities (which finance or finance and operate social care including home-help services) [3]. The organisation of geriatric medicine varies across countries. In Sweden it has been a recognized specialty since 1969. Specialized geriatric inpatient care (hereafter also referred to as care at geriatric department) is offered to all individuals, primarily over 65 years of age, with acute or chronic conditions relating to higher age as well as for short-term rehabilitation after inpatient care for medical or surgical conditions [4]. The individual’s well-being is dependent on the quality of the communication between these stakeholders and the continuity in healthcare [5]. Altogether, older adults in need of geriatric care are more than others in need of a well-functioning clinical pathway, regardless of who finances and delivers it.

Readmission rate over time is an indicator that enables understanding of the performance of the healthcare system and for the adequacy of care towards the individual geriatric patient. It is commonly used in studies on quality of care [6, 7]. A readmission is not possible to label as being due to malpractice during the previous admission or not; readmission after inpatient care at a geriatric department may for several reasons be inevitable. A high readmission rate could be due to organizational shortcomings as well as a tendency for an individual to seek care to a greater extent than necessary. Foremost, however, readmission may be due to the progress of an individual’s disease and a lowered health status [8]. There are several studies assessing the risk for readmission in older adults, and most studies focus on the risk in individuals with particular conditions or after particular interventions [9]. Multimorbidity is generally present in a majority of the older adults in need of geriatric care. Prevalence estimates of multimorbidity (defined as 2 or more co-existing chronic conditions) in individuals above 65 years of age vary between 10–15% [10] and 72% [11] for estimates of the general population. There are even higher numbers estimated based on individuals in need of primary care (as much as 98% in a study of a Danish population) [12], and for the individuals living in nursing homes (82% in a Dutch population) [11] respectively. In previous research, higher number of diagnoses has been found to correlate with higher risk for readmission [13]. In addition, the presence of both frailty and polypharmacy is associated with increased risk for readmission [14], and polypharmacy itself has been found to be a risk factor for readmission [15]. There is, however, little concluded on all-cause readmission rates and associated risk factors specifically for older adults in need of geriatric care.

Readmissions are generally very costly, in monetary terms as well as for the individual patient’s safety and health (also since the risk of an event or injury in general is higher at a healthcare institution than at home) [16]. There are several European examples where reduction in readmission rates is a prioritized goal, e.g. in France [17] and in Sweden [18]. There are also reimbursement-related penalties for hospitals and health systems in the US that show higher rates of readmissions than expected for particular conditions, as well as in the UK for readmission after a previous elective admission [6, 16]. It is therefore desirable to profile the groups that become readmitted, and to identify risk factors associated with readmissions. Only by understanding readmissions, their reasons and drivers, it is possible to ensure quality of care for these individuals as well as a chance to improve their situation.

The primary aim of this study was to assess inpatient readmissions during the first three months after discharge from geriatric inpatient care, regarding the main diagnosis at readmission and frequency of readmission. The secondary aim was to analyze association of selected factors (concerning demography, morbidity, physical function, risk screening, care process and socioeconomics) to any readmission as well as to repeated readmissions. Increased knowledge regarding associated factors could possibly guide clinical practitioners during geriatric inpatient care as well as in outpatient care after discharge and in collaboration with home-care services, to understand whether patients are at increased future risk and what to focus on in their secondary prevention activities.

## Materials and methods

### Study setting, population and data sources

The study includes all individuals admitted for inpatient care at any of the three geriatric departments operated by the Stockholm Region during 2016, and who lived in the Stockholm County throughout the three months following discharge (according to registrations made in the region’s administrative data and Statistics Sweden’s population database). The three departments for inpatient geriatric care are located as parts of three different hospital facilities, none with a separate emergency department. Staff includes specialized geriatricians, nurses and other health personnel trained for the geriatric patient. No study-specific alignment of process has been performed. However, the reources offered are supposed to be equivalent between the departments as they are all situated within the formal Stockholm healthcare region.

First, the last admission during 2016 at any of the three departments was considered to be the individual’s index admission. Second, all subsequent admissions were considered as readmissions, independent of reason for readmission or readmitting department, and the follow-up period was three months from the index admission discharge. Data were extracted from the electronic health records at the geriatric departments as well as from the Stockholm region’s administrative data warehouse (VAL), and linked on patient level to sociodemographic data from Statistics Sweden by using the Swedish national personal identification number. The study protocol was approved by the Regional Ethical Review board in Stockholm (reference numbers 2013/1620-31/2 and 2018/247-32). Due to the register based design of the study, informed consent was not collected, a procedure that was approved by the Regional Ethical Board in Stockholm and the health care authorities. The study population is the same as previously described by Rydwik and colleagues (2016 cohort) [19].

### Study variables

#### Outcomes

Readmission after discharge from a geriatric department was studied within three different time intervals; readmission within 10 days, within 11–30 days and within 31–90 days, respectively, after discharge, consistent with previous literature [9, 13, 20–23]. Consideration was taken to whether a new admission at another department during the day of discharge was a readmission (if the patient was discharged to home and/or admitted from home), or only as a transfer to another department (if the patient was discharged to another department and, there, registered as admitted from another department) and hence not considered a readmission. This was done via assessment of regional standard codes used for registering discharge destination and origin at admission. All readmissions included admissions at geriatric as well as non-geriatric departments, and no exclusions were made based on origin (e.g. nursing home).

Main diagnosis at readmission was also assessed, descriptively, and based on the three-digit level of the ICD-10 code.

#### Covariates

The selection of covariates for regression analysis was made based on previous studies and data availability. All risk factors have been assessed in previous research but not simultaneously as within this study. The set of covariates included several categories of patient characteristics (demography, morbidity-related factors, physical function, risk screening, and socioeconomic status) as well as care process indicators. A few covariates were transformed to binary variables, details presented above.

*Demographic factors* were sex and age. *Morbidity-related factors* included information on comorbidity (the number of diagnoses registered at discharge) and polypharmacy, both from medical records for the index admission. Polypharmacy was defined as having a prescription of five or more different medications (for continuous intake, at discharge), this numerical definition being the most common one in previous research [24].

*Physical function* indicators included the Barthel Activities of Daily Living (ADL) Index and the Rivermead Mobility Index (RMI). Barthel (ordinal scale, 0–100) was included as it is used to measure physical performance related to activities of daily living including dimensions such as walking, dressing, bathing, and was modeled as a continuous variable [25]. RMI is an instrument used for measuring mobility and transfer but have previously shown high correlation with Barthel [26]; a high score on either Barthel or RMI indicate a higher level of independence. For both RMI and Barthel the values at admission were used as these variables had significantly higher coverage rate than registrations made later during the index inpatient episode.

*Risk screening* included the Downton Fall Risk Index, the Norton pressure ulcer risk screening score and the Mini Nutritional Assessment (MNA) score. Downton, Norton and MNA were modeled as binary variables based on previous research and cut-off levels used in clinical practice; high risk of fall (Downton ≥3 [27]), risk of developing pressure ulcer (Norton≤20 [28]), and risk of malnutrition (MNA≤11 [29]). The risk screening was performed by trained healthcare personnel at each of the three departments during the index admission. A sensitivity analysis was performed where the original risk screening scores were used as covariates instead of the binary variables.

*Socioeconomic status* (SES) consisted of the patient’s highest educational level, region of birth and living situation. Educational level was modeled with five categories. The reason for this categorization was primarily to differentiate between primary and lower secondary school. The five levels were primary (<9 years), lower secondary (9–10 years), upper secondary (2–3 years), tertiary (1–3 years), and higher tertiary (Master degree or higher education). Region of birth (Sweden, other Nordic country, other European country, or the rest of the world) and living situation (living alone or cohabiting) were also included. Descriptive statistics of income is presented but was not used in regression analysis as educational level was expected to capture the same dimension of any socioeconomic gradient.

*Care process* indicators included length of stay (modeled as continuous variable) and discharge to ordinary home (modeled as binary variable). Ordinary home stands for the individual’s private residence and does not include care home or other temporary or permanent care facility.

### Statistical analysis

Descriptive statistics at baseline are presented for the study population as a whole and for readmitted and non-readmitted separately. The average levels for admitted (anytime during 90 days after discharge) versus non-admitted were compared using a chi-square test for categorical variables and a pooled t-test for continuous variables (assuming equal variances for the study variables between the groups). Also, a descriptive analysis of main diagnosis at readmission was performed.

Multivariate logistic regression analyses were performed for four different outcomes to study their associated factors and potential drivers; any readmission within time intervals 0–10 days after discharge, 11–30 days and 31–90 days respectively, as well as having had more than one readmission during days 0–90 (yes/no). Individuals with a 0–10 day readmission were not included in the analysis of 11–30 day readmission, nor were individuals with a 0–10 day readmission or an 11–30 day readmission included in the analysis of 31–90 day readmissions. Before including both Barthel ADL Index and RMI as independent variables, the correlation between the two variables (Pearson’s correlation coefficient, *r*) in the study population was computed to understand whether the two factors should be considered independent or not (cut-off set at r>0.7). Analysis of correlation between Barthel and RMI in this study population showed a Pearson coefficient of 0.80. Therefore, RMI was not included as independent factor alongside Barthel in the regression analysis.

Analysis of risk for readmission included only surviving individuals at each point in time. Duplicates were identified and deleted (based on participant personal identity number, admission date and date of discharge).

## Results

The study population consisted of 8,071 patients. The number of observations included in the regression analyses amounted to 6,632 (days 0–10), 6,061 (days 11–30), 5,339 (days 31–90), and 5,988 (several readmissions during days 0–90) individuals respectively, to include only survivors and observations with complete data for all explanatory variables (study population flowchart available in the additional files, S1 Fig). The study population (Table 1) consisted of 63% women, and the average age at index admission was 83.5 years. Twenty-two percent had completed primary school as highest educational level, 13% had completed lower secondary school, and 10% had completed higher tertiary education.

**Table 1:** Baseline patient characteristics of patients admitted to geriatric department during 2016.

		All			Not readmitted (days 0–90)		Readmitted (days 0–90)		
		Proportion or mean	SD	N	Proportion or mean	N	Proportion or mean	N	P value
**Demography**	**Sex, women**	63%		8071	65%	5982	56%	2089	0.000
	**Age**	83.5	8.2	8071	83.7	5982	83.0	2089	0.003
**Morbidity-related factors**	**Number of diagnoses** (at discharge from index admission)	4.7	1.8	8071	4.5	5982	5.0	2089	0.000
	**Polypharmacy (≥5)**	83%		8071	81%	5982	88%	2089	0.000
	**Pharmaceuticals ATC group 1**	8.6	4.2	7980	8.3	5899	9.5	2081	0.000
**Physical function**	**Barthel’s ADL index**	52.1	27.0	7216	52.3	5351	51.4	1865	0.200
	**RMI**	5.2	3.7	7370	5.2	5487	5.1	1883	0.471
**Risk screening**	**Fall risk, Downton value**	4.1	1.6	7967	4.1	5907	4.2	2060	0.016
	**Fall risk, Downton ≥3**	86%		7967	85%	5907	88%	2060	0.004
	**Pressure ulcer, Norton value**	22.0	3.4	7943	22.0	5899	21.9	2044	0.049
	**Pressure ulcer, Norton ≤20**	29%		7943	28%	5899	31%	2044	0.007
	**Malnutrition, MNA value**	8.8	2.6	7896	8.9	5864	8.6	2032	0.000
	**Malnutrition, MNA ≤11**	83%		7896	83%	5864	86%	2032	0.001
**Care process**	**Discharge to ordinary home**	76%		8071	77%	5982	74%	2089	0.032
	**Index length of stay**, at geriatric department	9.1	5.7	8071	9.0	5982	9.2	2089	0.176
**Socio-economic factors**	**Living alone**	61%		8058	61%	5972	60%	2086	0.229
	**Highest educational level**			7818		5797		2021	0.323
	Primary	22%			22%		22%		
	Lower secondary	13%			13%		12%		
	Upper secondary	36%			36%		37%		
	Post-secondary	19%			20%		19%		
	Higher post-secondary	10%			9%		10%		
	**Disposable income**, yearly, SEK	287 809		8065	287 360	5977	289 080	2088	0.906
	**Region of birth**			8064		5976		2088	0.981
	Sweden	83%			83%		83%		
	Other Nordic country	7%			7%		7%		
	Other Europe	7%			7%		7%		
	Outside Europe	3%			3%		3%		
	**Marital status**			8065		5977		2088	0.166
	Married	29%			29%		30%		
	Widowed	40%			41%		38%		
	Never married	10%			10%		10%		
	Divorced	21%			20%		22%		

There were significant differences between the sexes regarding age (84.3 years in women, 82.1 in men; p = 0.000), number of diagnoses at discharge from index admissions (4.5 in women, 4.9 in men; p = 0.000), and proportion discharged to home (77% in women, 74% in men; p = 0.002). Furthermore, all socioeconomic indicators differed between the sexes (p = 0.000). Men generally had higher formal educational level and higher disposable income (on average 26% higher), and a higher proportion of women was widowed (51% compared to 23% of men) and living alone (70% compared to 44% of men).

**Fig 1:**
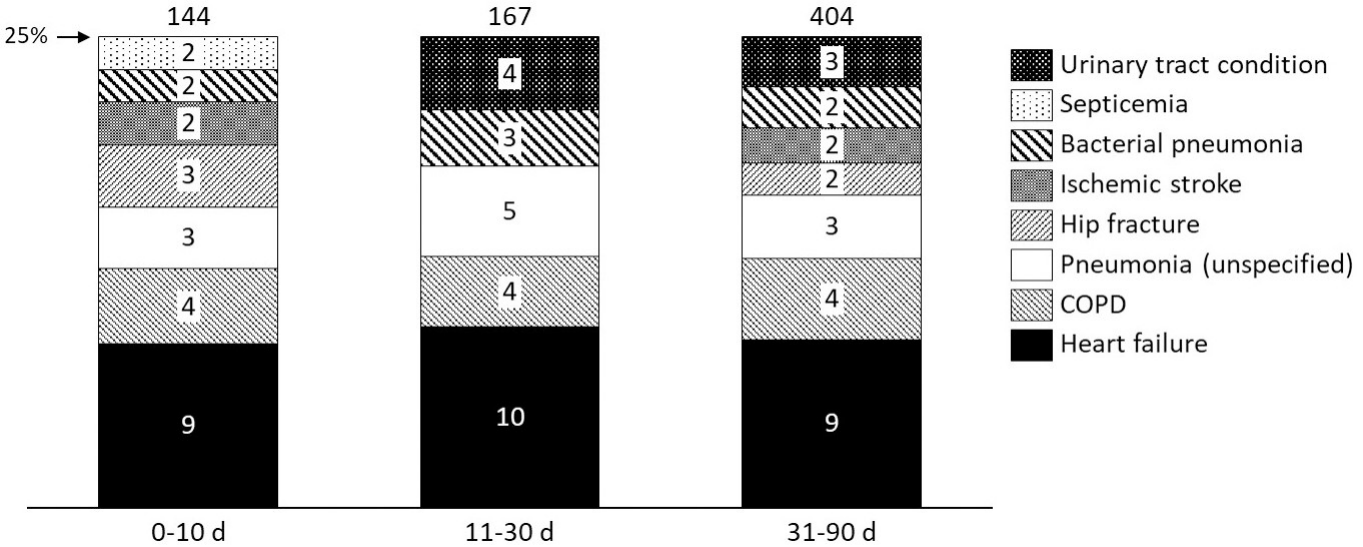
Proportion per main diagnosis for all readmissions within selected time intervals after discharge. The numbers in the bars represent percentage of total number of readmissions during the interval (totaling 25% per interval), and the number on top of the bars represents the number of readmissions during each interval with the main diagnoses included in the bars.

The patients were relatively evenly distributed amongst the three geriatric departments; 35.8% (2,891 patients), 26.6% (2,146 patients) and 37.6% (3,034) respectively. Fig 1 illustrates the most common main diagnoses at readmission over the three time intervals studied. For each interval, the most common main diagnoses that made up 25% of the readmissions are presented.

Main diagnosis at readmission remained similar during the time intervals studied; the most common one at readmission was heart failure for all three. At second place were conditions relating to chronic obstructive pulmonary disease (COPD), and thereafter unspecified pneumonia (unspecified pneumonia and bacterial pneumonia together were the second most common main diagnosis at readmission for all three intervals). Main diagnoses at readmission were as fragmented in the shorter time interval, within 10 days after discharge, as in the longer, within 31–90 days after discharge. Top 10 diagnoses for readmission within 10 days after discharge made up 29.6% of the readmissions, and the equivalent numbers were 33.2% during days 11–30 and 29.0% during days 31–90. The exact diagnoses differed somewhat between the time intervals; out of the top 10 during days 0–10, eight were part of the top 10 during days 31–90.

Table 2 presents readmission rates for the different time intervals studied. The proportion of readmitted individuals per day was continuously lower after discharge; it was higher during the first interval compared to the second, and higher during the second interval compared to the third. The proportion of readmitted individuals varied depending on how discharge and later same-day admission was treated; considering all discharges and new admissions the same day as actual readmissions implied a ten-day readmission rate of 8.3%. The proportion of readmissions with same main diagnosis as the index admission was similar throughout the selected time intervals (10.0–16.0%). More than one out of four (25.1%) was readmitted during the first three months after discharge from a geriatric department. Of those that survived three months after discharge, 6.9% were readmitted more than once.

**Table 2:** Readmission rates and proportions with same main diagnosis as during index admission.

	Proportion readmitted survivors within the time interval (cumulative proportion for second and third interval in parentheses)	Proportion of readmissions with same main diagnosis as during index admission
Readmission within 10 days	6.5% (7.0% of all, survivors and deceased)	16.0%
Readmission days 11–30 after index admission	7.7% (12.6% days 0–30)	11.1%
Readmission days 31–90 after index admission	15.9% (25.1% days 0–90)	10.0%
Several readmissions during days 0–90	6.9% (7.2% of all, survivors and deceased)	

Tables 3 and 4 present the coefficients from regression analysis of readmission within the selected time intervals.

**Table 3:** Risk factors for readmission within selected time intervals. Coefficients and confidence intervals from multivariate logistic regression analysis.

		Readmitted within 10 d.		Readmitted d. 11–30		Readmitted d. 31–90	
		(n = 6 632)		(n = 6 061)		(n = 5 339)	
		OR	95% CI	OR	95% CI	OR	95% CI
Demography	**Sex, women**	0.721***	0.583; 0.893	0.693***	0.561; 0.857	0.715***	0.603; 0.848
	**Age**	0.974***	0.962; 0.986	0.998	0.985; 1.010	0.993	0.983; 1.003
Morbidity-related factors	**Number of diagnoses** (per diagnosis at discharge from index admission)	1.097***	1.034; 1.163	1.143***	1.078; 1.211	1.178***	1.124; 1.235
	**Polypharmacy**	1.137	0.840; 1.540	1.284	0.943; 1.749	1.489***	1.160; 1.912
Physical function	**ADL index**, Barthel	1.005***	1.001; 1.010	1.003	0.999; 1.008	1.000	0.996; 1.004
Risk screening	**Fall**, Downton ≥3	1.174	0.851; 1.620	1.028	0.758; 1.395	0.977	0.767; 1.244
	**Pressure ulcer**, Norton ≤20	1.257	0.969; 1.631	1.434***	1.108; 1.857	0.922	0.742; 1.146
	**Risk of malnutrition**, Mini Nutritional Assessment ≤11	1.239	0.918; 1.673	1.155	0.870; 1.533	1.393***	1.110; 1.749
Care process	**Discharge to home**	0.509***	0.400; 0.647	1.132	0.858; 1.493	0.829	0.668; 1.030
	**Index length of stay** (per day, at geriatric dept)	0.996	0.976; 1.016	0.996	0.976; 1.017	0.992	0.975; 1.009
Socio-economic factors	**Living alone**	1.169	0.943; 1.449	0.984	0.798; 1.214	1.080	0.912; 1.280
	**Highest educational level**						
	Primary						
	Lower secondary	1.054	0.737; 1.507	0.872	0.602; 1.264	0.878	0.659; 1.169
	Upper secondary	1.037	0.788; 1.364	1.086	0.832; 1.418	0.935	0.754; 1.160
	Post-secondary	0.950	0.688; 1.312	0.917	0.667; 1.261	1.018	0.797; 1.300
	Higher post-secondary	1.047	0.711; 1.540	1.130	0.779; 1.638	1.004	0.741; 1.360
	**Region of birth**						
	Sweden						
	Other Nordic country	0.841	0.556; 1.271	1.194	0.829; 1.722	1.077	0.797; 1.454
	Other European	0.973	0.637; 1.486	1.033	0.681; 1.567	0.928	0.658; 1.308
	Outside Europe	0.592	0.274; 1.279	1.177	0.656; 2.111	1.118	0.691; 1.809
*c-statistic*		*0*.*620*		*0*.*612*		*0*.*619*	

Note:

*** denotes significant association with outcome on a 5% significance level (in multivariate analysis including all independent variables included in the table).

**Table 4:** Risk factors for more than one readmission during the first 90 days after discharge. Coefficients and confidence intervals from multivariate logistic regression analysis.

		Readmitted >1 time within 90 d.	
		(n = 5 988)	
		OR	95% CI
Demography	**Sex, women**	0.680***	0.547; 0.846
	**Age**	0.974***	0.961; 0.986
Morbidity-related factors	**Number of diagnoses** (per diagnosis at discharge from index admission)	1.185***	1.116; 1.259
	**Polypharmacy**	1.362	0.977; 1.899
Physical function	**ADL index**, Barthel	1.008***	1.003; 1.013
Risk screening	**Fall**, Downton ≥3	1.226	0.886; 1.695
	**Pressure ulcer**, Norton ≤20	1.400***	1.061; 1.847
	**Risk of malnutrition**, MNA ≤11	0.988	0.748; 1.305
Care process	**Discharge to home**	0.758	0.574; 1.001
	**Index length of stay** (per day, at geriatric dept)	0.995	0.974; 1.017
Socio-economic factors	**Living alone**	0.957	0.770; 1.188
	**Highest educational level**		
	Primary		
	Lower secondary	0.983	0.675; 1.433
	Upper secondary	1.062	0.797; 1.414
	Post-secondary	0.946	0.677; 1.323
	Higher post-secondary	1.003	0.674; 1.493
	**Region of birth**		
	Sweden		
	Other Nordic country	0.939	0.623; 1.416
	Other European	0.963	0.619; 1.497
	Outside Europe	0.770	0.386; 1.536
*c-statistic*		*0*.*641*	

Note:

*** denotes significant association with outcome on a 5% significance level (in multivariate analysis including all independent variables included in the table).

Demographic factors were significantly associated with readmission. Female sex was associated with lower risk of readmission during all three time intervals, as well as with being readmitted more than once during the first three months after discharge. Age was associated with risk for readmission during the first ten days and with the risk for several readmissions; the higher the age, the lower the risk.

Morbidity-related factors were associated with readmission. Registered comorbidity (number of diagnoses) during index admission was consistently associated with the outcomes, for all three time intervals as well as regarding risk of several readmissions; the higher the number of diagnoses, the higher the risk of readmission. Polypharmacy was positively associated with risk of readmission during the last time interval, days 31–90.

The association with physical function and risk screening factors varied depending on time interval after discharge. Higher Barthel score, i.e. better physical function, was associated with higher risk of readmission during the first ten days and with the risk of being readmitted more than once. Fall risk according to Downton was not associated with risk for readmission. Risk of developing pressure ulcer (Norton) during the index admission was positively associated with readmission during days 11–30 after discharge as well as with being readmitted more than once. Risk of malnutrition (based on MNA score) was positively associated with readmission during days 31–90 after discharge. The reasons for why risk of pressure ulcer versus malnutrition may impact readmission differently over time are not clear. However, if being readmitted within 90 days, any risk of pressure ulcer could imply a quicker need for additional inpatient care, whilst malnutrition becomes clinically manifestable only after a longer period of time, thus presenting with readmission only after 30 days from discharge.

Care process was partially associated to risk for readmission. Discharge to ordinary home was negatively associated with readmission during the first ten days, i.e. being discharged to ordinary home instead of other facility was associated with a short-term decrease in risk for a new admission.

Socioeconomic status—the individual’s educational level, origin and cohabiting status—did not add to the predictability of readmission during the selected time intervals.

Results from sensitivity analyses performed (regression analysis including deceased individuals as well as a regression analysis with risk screening scores used as continuous variables) are presented in a supplementary file (S1 File).

## Discussion

### General findings

Thirteen percent of patients with a previous geriatric inpatient admission were readmitted during the first month after discharge (in line with previous assessments made by the Swedish National Board of Health and Welfare [30]), and 25% were readmitted within the first three months. The most common formal reasons for readmission, the main diagnoses, were similar throughout the three time intervals studied. Main diagnosis at readmission generally related to a chronic condition (heart failure, COPD) or a potentially acute exacerbation of the individual’s health status (pneumonia, septicemia). In previous research, chronic conditions have been presented as a dominating reason for admission and readmission [31, 32]. This fact points to a potentially suboptimal management of patients with these chronic conditions in outpatient care; by a proactive strategy, it could be possible to bypass the need for a readmission and improve the care within outpatient care with less costly but as effective secondary preventive measures. The appropriateness of a readmission differs depending on reason; if it is medically motivated it cannot be considered avoidable at the time of readmission. Still, in that same case, there may be room for improvement in the process leading up to the readmission, during the index admission as well as during the time after discharge. Information on readmission history may also be a valuable factor to predict future risk for morbidity and death [33]. Today, the access to such information, including historic health records, may depend on how healthcare is organized, or on the integration of the local electronic health records system—which is accentuated in the Stockholm region. The coordination of efforts for older and disabled adults may also be particularly challenging in the Stockholm context as healthcare provided at home is financed by the region (although operated by several different private providers), separate from the municipality-financed care homes and (non-healthcare) home-help services.

Several of the analyzed factors were significantly associated with risk for readmission. Factors positively associated with higher risk for readmission included demography (male sex, lower age), morbidity-related factors (higher comorbidity and polypharmacy), and care process (discharge to other institution than ordinary home). Furthermore, several risk screening factors were associated with risk for readmission, described in more detail below. A core question related to the present study is whether the risk for readmission is associated with clinically relevant, legitimate factors, hence if a difference in readmission risk should be considered warranted. For example, the number of individual diagnoses, or multimorbidity, could be considered a legitimate factor of clinical relevance regarding the risk for need of additional inpatient care, as possibly age and ADL (Barthel index score). The clinical feasibility of a readmission is however as much dependent on the setup and organization of the healthcare system. For example, primary-care teams specialized in geriatric care could help mitigating readmissions, and existence of such resources could likely have an impact on readmission rates.

### Risk factor assessment

Age was associated with partially increased risk of admission; during days 0–10 and regarding more than one readmission the first 90 days after discharge. Interestingly, higher age was associated with lower risk for readmission. Several previously published studies have found no association between age and risk for readmission, of which a few also were able to adjust for socioeconomic status and risk screening factors in addition to sex and age [34–37]. In this study several factors of significance to the individual were simultaneously adjusted for. Regarding age and the inherent higher risk of living at nursing home with higher age, previous research has pointed to age-independent differences depending on nursing home performance [38], i.e. appropriate competence and capability. Logically, younger individuals in general do have a longer life expectancy than older, and hence a pre-defined higher probability to get admitted again, in a long-term perspective. Sex was consistently associated with readmission risk, also when simultaneously controlling for other factors; women ran a lower risk for readmission disregarding the time interval studied. Previous research has not unanimously pointed in one direction; research has shown higher readmission for men (for e.g. COPD patients) [39] and some for women (e.g. after acute myocardial infarction) [40]. Differences in readmission between women and men have also been shown to vary between age groups [41].

Number of diagnoses registered during the index admission was positively associated with risk of readmission, for all time intervals. The same pattern has been reported in previous studies, especially for individuals afflicted with multiple chronic conditions [13]. In the present study, polypharmacy was simultaneously associated to higher risk of readmission (days 31–90), and polypharmacy has been reported to increase the risk for readmission in a similar population [42]. It is feasible to believe that any pharmaceutical interaction effects would come at a later stage and not immediately after discharge. However, no analysis was made to take different substances into account, only the total numbers, and it is not known what additional medications that were prescribed during the index admission versus before.

Physical function and some risk screening factors, although not fall risk, were associated to readmission risk, but the pattern looked different depending on time interval studied. Higher Barthel index score was associated with higher risk of readmission during the first ten days and with higher risk of several readmissions during the first 90 days. This may seem counterintuitive, i.e. with a better physical function it was more likely to be readmitted. However, individuals with worse physical function, hence possibly more frail, are generally more likely to be discharged to a nursing home, and living at a nursing home may alter the risk for readmission in both directions compared to living in ordinary home depending on nursing-home performance, as pointed out in previous studies [38]. Differences in skill and competence levels between nursing homes have also been shown to have an impact [43]. It is worth noting the competing risk due to death; the mortality rates in the more frail and less independent individuals living in nursing homes are higher [38]. Norton score, risk to develop pressure ulcer, and MNA score, risk for malnutrition during index admission and a possible indicator of frailty, came out as significant for some of the time intervals studied; Norton was positively associated with readmission during days 11–30 and with risk of several admissions, and risk of malnutrition was positively associated with readmission during days 31–90. Fall risk (Downton, present in 86% of the study population) was not associated with risk of readmission, which contradicts previous research [8, 22], although a meta-analysis has pointed to the ambivalence of using fall risk tools for prediction as correct useability is highly dependent on study design [44]. In a sensitivity analysis, the three risk screening factors were modeled as continuous variables instead of being dichotomized as in the original analysis, with similar results regarding the statistical association with outcomes.

The association between care process indicators and readmission was limited. Discharge to home was negatively associated with risk for readmission during the first 10 days after discharge. Length of stay was not associated to risk of readmission. In previous research this has been put forward as a potentially important determinant for readmission risk, although the explanatory value of the index length of stay has been shown to decrease with a broader range of explanatory factors [8].

Interestingly, socioeconomic status did not increase the predictability of readmissions. Previous research on readmission in a cohort of older (65 years of age or older) adults has pointed to the absence of a socioeconomic gradient [15]. Sensitivity analysis included regression analysis with interaction terms of e.g. sex and educational level and still no such association was found. Statistical Chi-squared tests showed that women had significantly lower educational level (p = 0.000), and lower income (p = 0.000). Adjusting for both sex and socioeconomic status, as in the present study, any systematic socioeconomic risk differences between sexes is likely captured by the sex category variable. Also, the Stockholm region has shown significant differences in socioeconomic status between geographic areas, which may also impact the analysis in the present study as the study population is based on three geriatric departments serving specific geographical areas of Stockholm [45].

### Limitations and strengths

There are a couple of limitations in the present study. Informal care has not been accounted for in this study and it is known from previous research that this may have significant impact on the degree of resource utilization in the healthcare system [46]. Furthermore, municipality-financed care is not included, which implies no information regarding post-discharge care at care home or with non-medical home-help services. There is an uncertainty regarding the patients’ actual comorbidity as only registrations made during index admission were included, and likely the predictability would increase with a longer history of medical records to adjust for. The pharmacological utilization is also associated with uncertainty as only pharmaceuticals found in previous patient records or registered during the index admission stay were included. It is not known whether there have been additional admissions to any of the geriatric departments beyond the period of six months after the index admission, and any previous history of readmission is not taken into account; as previously described [17], a history of admissions is a risk factor for additional admissions. Furthermore, and has been mentioned, the competing risk due to death is not negligible for this study population; 14% died within 90 days after discharge. This may introduce a bias as readmission risks were analyzed only for survivors at the end of each time interval. To understand the implications of such a potential bias, sensitivity analysis was performed via performing equivalent multivariate regression analyses but also including individuals that were deceased at the end of follow-up. Results from these analyses showed consistent patterns as in the original analyses; the only differences were that polypharmacy presented as significant for all intervals, and that discharge destination was significantly associated also with readmission days 11–30.

To recognize the strengths of this study, the size of the study population should be mentioned; it is large and covers all individuals who received inpatient care at the Stockholm-region run geriatric departments during a full year, 2016. Furthermore, individual-level socioeconomic information has been controlled for, along with key clinical factors as well as indicators on the care process. This was possible by linking individual-level data from several data sources (medical records, the regional administrative data warehouse, and national research registries from Statistics Sweden) via the personal identification number.

## Conclusions

One fourth of the individuals treated inpatient at a geriatric Stockholm region department was readmitted during the first three months after discharge. The most common main diagnoses for readmission were heart failure, COPD and pneumonia. Risk factors for readmission included age, sex, number of diagnoses at discharge, and to some extent polypharmacy and destination of discharge. Socioeconomic information did not seem to play a role regarding risk of readmission.

These findings stress the significant burden that readmission puts on the health system. Furthermore, it illustrates what factors that may be used to predict the readmission risk or to predict the need for higher attention in outpatient care. The presence of e.g. comorbidity, polypharmacy, and discharge to other institution than ordinary home, would support the anticipation that the individual will be readmitted during a three-month follow-up. Obviously, this could be practically translated into a higher attention to those individuals, to mitigate the need for inpatient care—also put forward in previous research [47]. Pro-active monitoring is necessary to enable reductions in readmission rates, and an integrated perspective from all stakeholders involved is essential to make it successful. Despite the desire to reduce readmission rate, there is likely an underlying, constant risk of readmission that has to be dealt with by the health system. Defining a new admission after previous inpatient care as a readmission has been done based on various perspectives in previous studies, and generally viewing all new admissions within a given time frame as readmissions seems to be one of the most common [6]. What would perhaps be more appropriate is a common standard for the definition of a readmission, whilst such a standard would need to be specific based on the morbidity studied [6]. For the focus of the present study, the time frame as delimiter seems appropriate.

## Supporting information

S1 Fig Flowchart of exclusions resulting in the study population. Out of 8 104 patients, only 8 091 were residents in Stockholm region at time of the index admission. Twenty of these 8 091 individuals moved out of Stockholm region sometime during follow-up (based on place of residency according to administrative information attached to outpatient care contacts); 8 071 individuals remained in the study population.
(JPG)

S2 Fig Flowchart of exclusions made for each regression analysis performed. (JPG)

S1 File Tables A1-A8 sensitivity analyses of the original multivariate regression analyses. Tables show results based on inclusion of deceased individuals (A1-A4) as well as based on using risk factor screening scores as continuous variables instead of binary variables (A5-A8).
(DOCX)

## Abbreviations

ADL: Basic activities of daily living
MNA: Mini Nutritional Assessment
RMI: Rivermead Mobility Index
VAL: Stockholm Regional Healthcare Data Warehouse
