# Supplementary figures and images for: Readmission within three months after inpatient geriatric care—Incidence, diagnosis and associated factors in a Swedish cohort
Source: PLoS One. 2021 Mar 22;16(3):e0248972. doi: 10.1371/journal.pone.0248972 (PMC7984622; doi:10.1371/journal.pone.0248972)

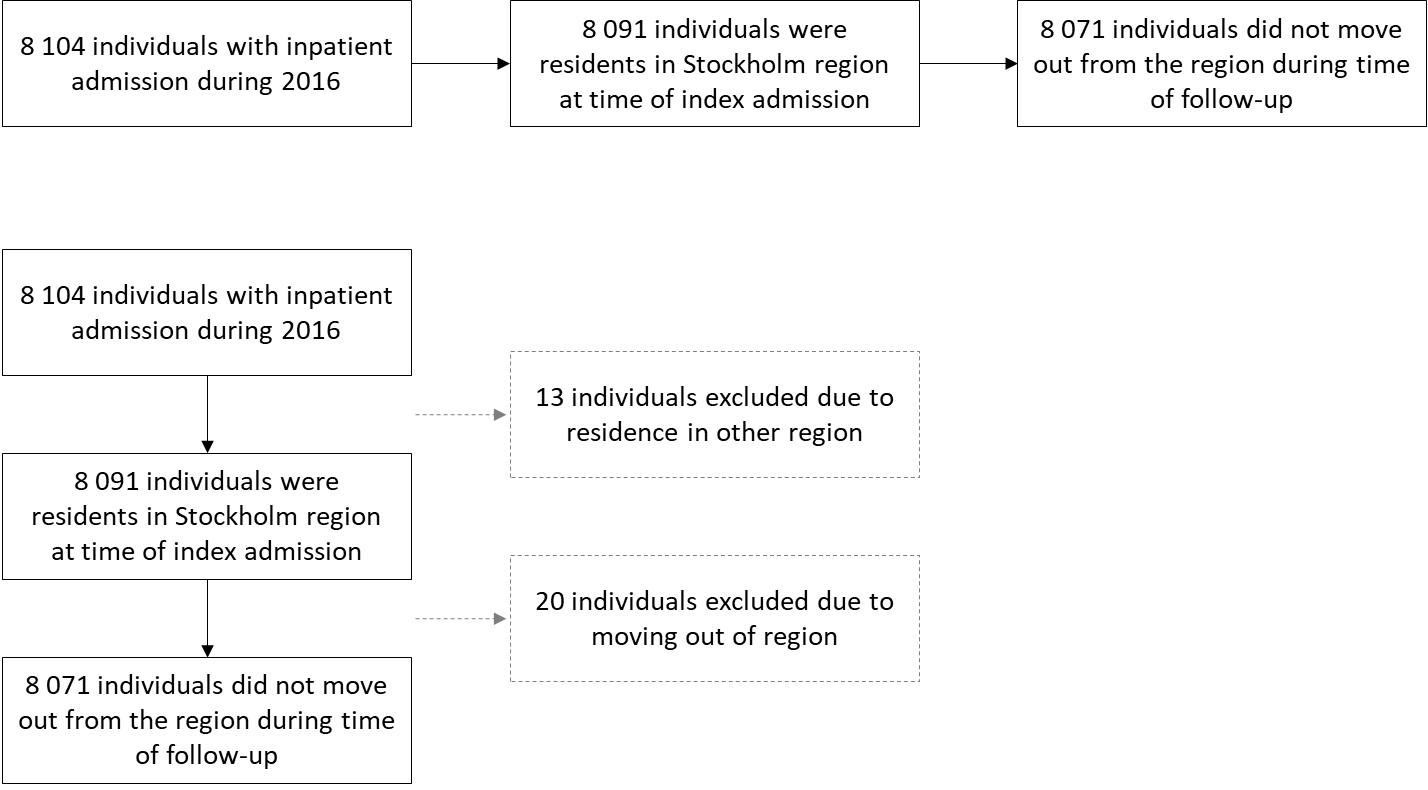

Supplement: S1 Fig — Out of 8 104 patients, only 8 091 were residents in Stockholm region at time of the index admission. Twenty of these 8 091 individuals moved out of Stockholm region sometime during follow-up (based on place of residency according to administrative information attached to outpatient care contacts); 8 071 individuals remained in the study population. (JPG) [file pone.0248972.s001.jpg]

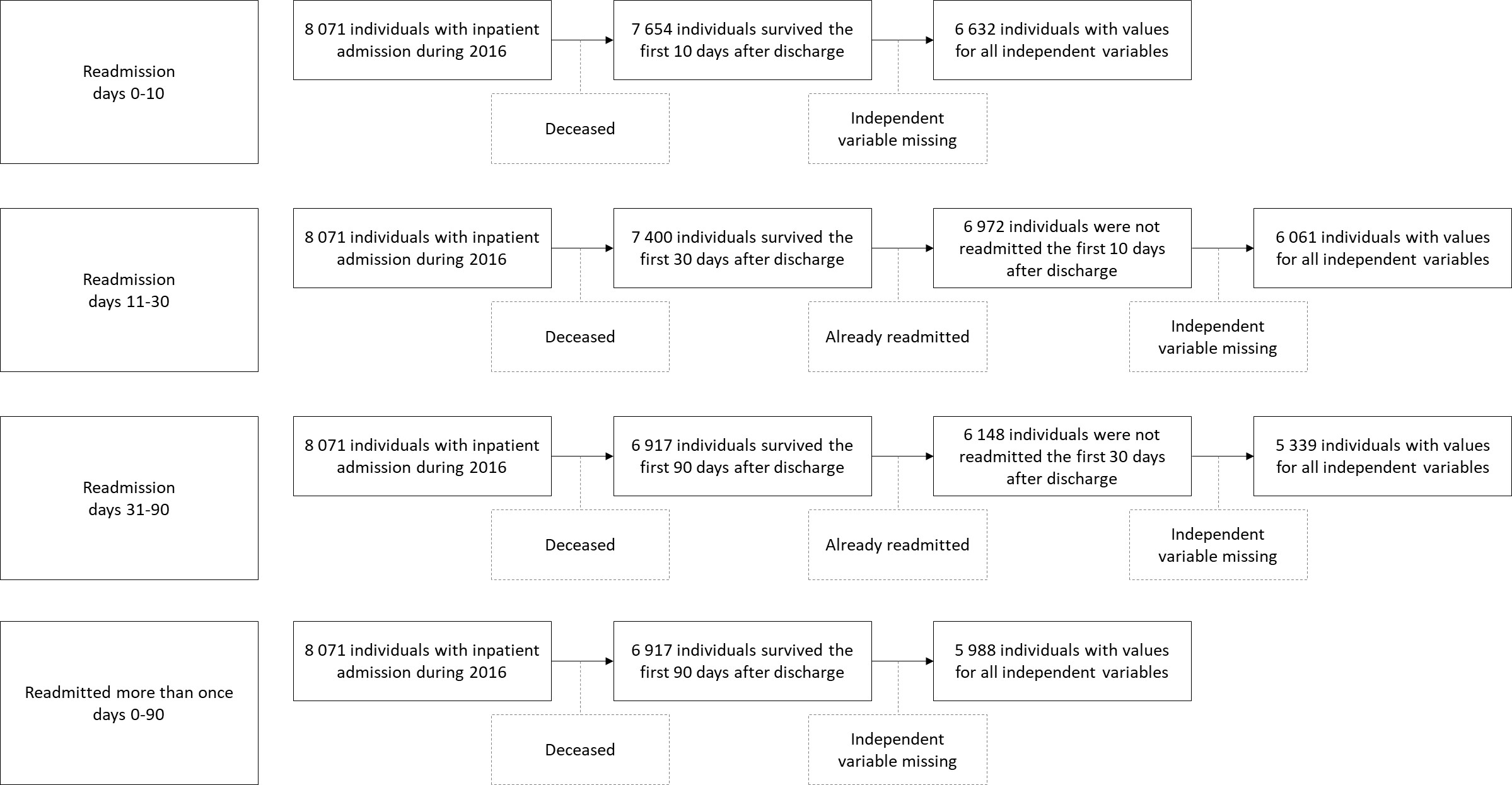

Supplement: S2 Fig — (JPG) [file pone.0248972.s002.jpg]
