# Supplementary material for: Readmission within three months after inpatient geriatric care—Incidence, diagnosis and associated factors in a Swedish cohort
Source: PLoS One. 2021 Mar 22;16(3):e0248972. doi: 10.1371/journal.pone.0248972 (PMC7984622; doi:10.1371/journal.pone.0248972)
Supplement: S1 File — Tables show results based on inclusion of deceased individuals (A1-A4) as well as based on using risk factor screening scores as continuous variables instead of binary variables (A5-A8). (DOCX) [file pone.0248972.s003.docx]

**S1 File – sensitivity analyses**

**Deceased included**

|  |  | Readmission in 0-10 days | |
| --- | --- | --- | --- |
|  | OR | 95% CI - lower | 95% CI - upper |
| **Sex, women** | 0.698 | 0.571 | 0.854 |
| **Age** | 0.977 | 0.965 | 0.988 |
| **Number of diagnoses** (per diagnosis at discharge from index admission) | 1.090 | 1.032 | 1.151 |
| **Polypharmacy** | 1.204 | 0.909 | 1.595 |
| **ADL index**, Barthel | 1.005 | 1.000 | 1.009 |
| **Pressure ulcer**, Norton <20 | 1.229 | 0.899 | 1.682 |
| **Risk of malnutrition**, MNA <11 | 1.216 | 0.952 | 1.554 |
| **Fall**, Downton >3 | 1.290 | 0.964 | 1.726 |
| **Discharge to home** | 0.514 | 0.411 | 0.643 |
| **Index length of stay (per day, at geriatric dept)** | 0.994 | 0.976 | 1.012 |
| **Living alone** | 1.115 | 0.912 | 1.364 |
| Higher post-secondary | 1.135 | 0.795 | 1.619 |
| Post-secondary | 0.935 | 0.689 | 1.270 |
| Upper secondary | 1.041 | 0.804 | 1.347 |
| Lower secondary | 1.013 | 0.718 | 1.429 |
| Other Nordic country | 0.527 | 0.244 | 1.138 |
| Other European | 1.019 | 0.690 | 1.505 |
| Outside Europe | 0.850 | 0.574 | 1.259 |

|  |  | Readmission days 11-30 | |
| --- | --- | --- | --- |
|  | OR | 95% CI - lower | 95% CI - upper |
| **Sex, women** | 0.668 | 0.546 | 0.818 |
| **Age** | 0.999 | 0.987 | 1.011 |
| **Number of diagnoses** (per diagnosis at discharge from index admission) | 1.128 | 1.068 | 1.191 |
| **Polypharmacy** | 1.485 | 1.101 | 2.003 |
| **ADL index**, Barthel | 1.003 | 0.998 | 1.007 |
| **Pressure ulcer**, Norton <20 | 1.043 | 0.777 | 1.401 |
| **Risk of malnutrition**, MNA <11 | 1.313 | 1.024 | 1.683 |
| **Fall**, Downton >3 | 1.106 | 0.846 | 1.446 |
| **Discharge to home** | 1.354 | 1.044 | 1.756 |
| **Index length of stay (per day, at geriatric dept)** | 0.997 | 0.978 | 1.016 |
| **Living alone** | 1.003 | 0.821 | 1.225 |
| Higher post-secondary | 1.089 | 0.761 | 1.558 |
| Post-secondary | 0.911 | 0.672 | 1.236 |
| Upper secondary | 1.092 | 0.847 | 1.407 |
| Lower secondary | 0.961 | 0.681 | 1.357 |
| Other Nordic country | 1.341 | 0.787 | 2.283 |
| Other European | 1.160 | 0.794 | 1.695 |
| Outside Europe | 1.227 | 0.866 | 1.737 |
|  |  | Readmission days 31-90 |  |
|  | OR | 95% CI - lower | 95% CI - upper |
| **Sex, women** | 0.763 | 0.650 | 0.895 |
| **Age** | 0.995 | 0.986 | 1.005 |
| **Number of diagnoses** (per diagnosis at discharge from index admission) | 1.150 | 1.101 | 1.200 |
| **Polypharmacy** | 1.617 | 1.284 | 2.035 |
| **ADL index**, Barthel | 1.001 | 0.997 | 1.004 |
| **Pressure ulcer**, Norton <20 | 0.943 | 0.752 | 1.182 |
| **Risk of malnutrition**, MNA <11 | 0.949 | 0.778 | 1.157 |
| **Fall**, Downton >3 | 1.401 | 1.127 | 1.741 |
| **Discharge to home** | 1.082 | 0.890 | 1.314 |
| **Index length of stay (per day, at geriatric dept)** | 0.994 | 0.980 | 1.009 |
| **Living alone** | 1.099 | 0.938 | 1.288 |
| Higher post-secondary | 0.984 | 0.741 | 1.306 |
| Post-secondary | 0.991 | 0.791 | 1.242 |
| Upper secondary | 0.896 | 0.736 | 1.091 |
| Lower secondary | 0.801 | 0.613 | 1.048 |
| Other Nordic country | 0.992 | 0.623 | 1.580 |
| Other European | 0.896 | 0.649 | 1.239 |
| Outside Europe | 1.069 | 0.807 | 1.415 |

|  |  | Readmitted several times during days 0-90 | |
| --- | --- | --- | --- |
|  | OR | 95% CI - lower | 95% CI - upper |
| **Sex, women** | 0.699 | 0.573 | 0.852 |
| **Age** | 0.978 | 0.966 | 0.989 |
| **Number of diagnoses** (per diagnosis at discharge from index admission) | 1.137 | 1.078 | 1.200 |
| **Polypharmacy** | 1.532 | 1.131 | 2.075 |
| **ADL index**, Barthel | 1.008 | 1.003 | 1.013 |
| **Pressure ulcer**, Norton <20 | 1.156 | 0.862 | 1.550 |
| **Risk of malnutrition**, MNA <11 | 1.354 | 1.056 | 1.734 |
| **Fall**, Downton >3 | 1.036 | 0.798 | 1.344 |
| **Discharge to home** | 0.977 | 0.765 | 1.248 |
| **Index length of stay (per day, at geriatric dept)** | 0.998 | 0.980 | 1.017 |
| **Living alone** | 0.982 | 0.807 | 1.196 |
| Higher post-secondary | 0.893 | 0.620 | 1.284 |
| Post-secondary | 0.864 | 0.641 | 1.165 |
| Upper secondary | 0.959 | 0.745 | 1.233 |
| Lower secondary | 0.974 | 0.699 | 1.357 |
| Other Nordic country | 0.643 | 0.324 | 1.275 |
| Other European | 0.976 | 0.658 | 1.448 |
| Outside Europe | 0.968 | 0.671 | 1.397 |

**Risk screening factors used as continuous variables**

|  | Readmission in 0-10 days | | |
| --- | --- | --- | --- |
|  | OR | 95% CI - lower | 95% CI - upper |
| **Sex, women** | 0.728 | 0.586 | 0.903 |
| **Age** | 0.974 | 0.962 | 0.987 |
| **Number of diagnoses** (per diagnosis at discharge from index admission) | 1.097 | 1.034 | 1.164 |
| **Polypharmacy** | 1.169 | 0.857 | 1.595 |
| **ADL index**, Barthel | 1.006 | 1.000 | 1.011 |
| **Pressure ulcer**, Norton score | 0.962 | 0.918 | 1.007 |
| **Risk of malnutrition**, MNA score | 1.007 | 0.960 | 1.056 |
| **Fall**, Downton score | 1.009 | 0.940 | 1.082 |
| **Discharge to home** | 0.501 | 0.392 | 0.639 |
| **Index length of stay (per day, at geriatric dept)** | 1.000 | 0.980 | 1.020 |
| **Living alone** | 1.191 | 0.958 | 1.480 |
| Higher post-secondary | 1.037 | 0.702 | 1.532 |
| Post-secondary | 0.956 | 0.691 | 1.323 |
| Upper secondary | 1.018 | 0.771 | 1.343 |
| Lower secondary | 1.047 | 0.731 | 1.498 |
| Other Nordic country | 0.622 | 0.288 | 1.346 |
| Other European | 0.997 | 0.652 | 1.523 |
| Outside Europe | 0.853 | 0.563 | 1.290 |

|  | Readmission days 11-30 | | |
| --- | --- | --- | --- |
|  | OR | 95% CI - lower | 95% CI - upper |
| **Sex, women** | 0.688 | 0.556 | 0.851 |
| **Age** | 0.997 | 0.984 | 1.010 |
| **Number of diagnoses** (per diagnosis at discharge from index admission) | 1.138 | 1.073 | 1.207 |
| **Polypharmacy** | 1.315 | 0.963 | 1.796 |
| **ADL index**, Barthel | 1.005 | 0.999 | 1.010 |
| **Pressure ulcer**, Norton score | 0.958 | 0.916 | 1.002 |
| **Risk of malnutrition**, MNA score | 0.949 | 0.906 | 0.995 |
| **Fall**, Downton score | 1.004 | 0.937 | 1.076 |
| **Discharge to home** | 1.178 | 0.889 | 1.561 |
| **Index length of stay (per day, at geriatric dept)** | 0.993 | 0.972 | 1.014 |
| **Living alone** | 0.975 | 0.790 | 1.204 |
| Higher post-secondary | 1.162 | 0.801 | 1.686 |
| Post-secondary | 0.895 | 0.649 | 1.233 |
| Upper secondary | 1.066 | 0.816 | 1.393 |
| Lower secondary | 0.857 | 0.591 | 1.242 |
| Other Nordic country | 1.124 | 0.613 | 2.060 |
| Other European | 1.027 | 0.672 | 1.569 |
| Outside Europe | 1.216 | 0.843 | 1.754 |

|  | Readmission days 31-90 | | |
| --- | --- | --- | --- |
|  | OR | 95% CI - lower | 95% CI - upper |
| **Sex, women** | 0.718 | 0.604 | 0.852 |
| **Age** | 0.994 | 0.983 | 1.004 |
| **Number of diagnoses** (per diagnosis at discharge from index admission) | 1.182 | 1.127 | 1.240 |
| **Polypharmacy** | 1.509 | 1.171 | 1.945 |
| **ADL index**, Barthel | 1.000 | 0.995 | 1.004 |
| **Pressure ulcer**, Norton score | 1.022 | 0.984 | 1.060 |
| **Risk of malnutrition**, MNA score | 0.954 | 0.919 | 0.990 |
| **Fall**, Downton score | 0.999 | 0.945 | 1.057 |
| **Discharge to home** | 0.840 | 0.674 | 1.046 |
| **Index length of stay (per day, at geriatric dept)** | 0.990 | 0.974 | 1.007 |
| **Living alone** | 1.094 | 0.922 | 1.298 |
| Higher post-secondary | 0.979 | 0.720 | 1.332 |
| Post-secondary | 1.033 | 0.808 | 1.321 |
| Upper secondary | 0.931 | 0.750 | 1.156 |
| Lower secondary | 0.877 | 0.658 | 1.169 |
| Other Nordic country | 1.155 | 0.713 | 1.870 |
| Other European | 0.929 | 0.657 | 1.315 |
| Outside Europe | 1.090 | 0.807 | 1.473 |

|  | Readmitted several times during days 0-90 | | |
| --- | --- | --- | --- |
|  | OR | 95% CI - lower | 95% CI - upper |
| **Sex, women** | 0.679 | 0.544 | 0.846 |
| **Age** | 0.972 | 0.960 | 0.985 |
| **Number of diagnoses** (per diagnosis at discharge from index admission) | 1.179 | 1.109 | 1.253 |
| **Polypharmacy** | 1.365 | 0.976 | 1.910 |
| **ADL index**, Barthel | 1.008 | 1.003 | 1.013 |
| **Pressure ulcer**, Norton score | 0.972 | 0.926 | 1.020 |
| **Risk of malnutrition**, MNA score | 0.967 | 0.921 | 1.015 |
| **Fall**, Downton score | 1.045 | 0.972 | 1.123 |
| **Discharge to home** | 0.774 | 0.584 | 1.024 |
| **Index length of stay (per day, at geriatric dept)** | 0.993 | 0.972 | 1.015 |
| **Living alone** | 0.950 | 0.764 | 1.181 |
| Higher post-secondary | 1.023 | 0.687 | 1.523 |
| Post-secondary | 0.937 | 0.670 | 1.313 |
| Upper secondary | 1.044 | 0.783 | 1.393 |
| Lower secondary | 0.971 | 0.666 | 1.415 |
| Other Nordic country | 0.799 | 0.400 | 1.593 |
| Other European | 0.940 | 0.599 | 1.475 |
| Outside Europe | 0.945 | 0.627 | 1.425 |
